# Development of the African Killifish as a New Model to Study Aging and Suspended animation

**DOI:** 10.1093/geroni/igaa057.2668

**Published:** 2020-12-16

**Authors:** Anne Brunet

**Affiliations:** Stanford School of Medicine, Stanford, California, United States

## Abstract

We have pioneered a new model organism for aging research, the naturally short-lived African killifish Nothobranchius furzeri. The African killifish lives in ephemeral pools of water in Africa, and has evolved a short life cycle adapted to this habitat. Its embryos can also resist drought until the next wet season in a state of ‘suspended life’. In laboratory conditions, the African killifish has a maximal lifespan of about 4-6 months, and is, so far, the shortest-lived vertebrate that can be bred in captivity. We have successfully transformed this natural short-lived vertebrate into a usable model organism for aging research, including de novo assembly of the genome and CRISPR-Cas9 mediated genome-editing. Our goal is to use this model to discover new principles underlying aging, longevity, and ‘suspended animation’ in vertebrates.

